# The New Ice Age of Musculoskeletal Intervention: Role of Percutaneous Cryoablation in Bone and Soft Tissue Tumors

**DOI:** 10.3390/curroncol30070495

**Published:** 2023-07-17

**Authors:** Nicolas Papalexis, Leonor Garbin Savarese, Giuliano Peta, Costantino Errani, Gianmarco Tuzzato, Paolo Spinnato, Federico Ponti, Marco Miceli, Giancarlo Facchini

**Affiliations:** 1 Diagnostic and Interventional Radiology Unit, IRCCS Istituto Ortopedico Rizzoli, 40136 Bologna, Italy; giuliano.peta@ior.it (G.P.); paolo.spinnato@ior.it (P.S.); federico.ponti@ior.it (F.P.); marco.miceli@ior.it (M.M.); giancarlo.facchini@ior.it (G.F.); 2 Department of Medical Imaging, Hematology and Clinical Oncology, Ribeirao Preto Medical School, University of Sao Paulo, Ribeirão Preto 14049-09, Brazil; leonorgs@alumni.usp.br; 3 Department of Orthopaedic Oncology, IRCCS Istituto Ortopedico Rizzoli, 40136 Bologna, Italy; costantino.errani@ior.it (C.E.); gianmarco.tuzzato@ior.it (G.T.)

**Keywords:** interventional radiology, metastatic neoplasms, orthopedic surgery, palliative medicine, cryoablation

## Abstract

In the rapidly evolving field of interventional oncology, minimally invasive methods, including CT-guided cryoablation, play an increasingly important role in tumor treatment, notably in bone and soft tissue cancers. Cryoablation works using compressed gas-filled probes to freeze tumor cells to temperatures below −20 °C, exploiting the Joule–Thompson effect. This cooling causes cell destruction by forming intracellular ice crystals and disrupting blood flow through endothelial cell damage, leading to local ischemia and devascularization. Coupling this with CT technology enables precise tumor targeting, preserving healthy surrounding tissues and decreasing postoperative complications. This review reports the most important literature on CT-guided cryoablation’s application in musculoskeletal oncology, including sarcoma, bone metastases, and bone and soft tissue benign primary tumors, reporting on the success rate, recurrence rate, complications, and technical aspects to maximize success for cryoablation in the musculoskeletal system.

## 1. Introduction

Interventional oncology has emerged as an increasingly crucial component of the multidisciplinary team, providing innovative and minimally invasive treatment approaches for various types of musculoskeletal tumors [1,2]. One such innovative technique, CT-guided cryoablation, is increasingly gaining recognition for its effectiveness, precision, and improved patient outcomes [3,4].

**Figure 1:**
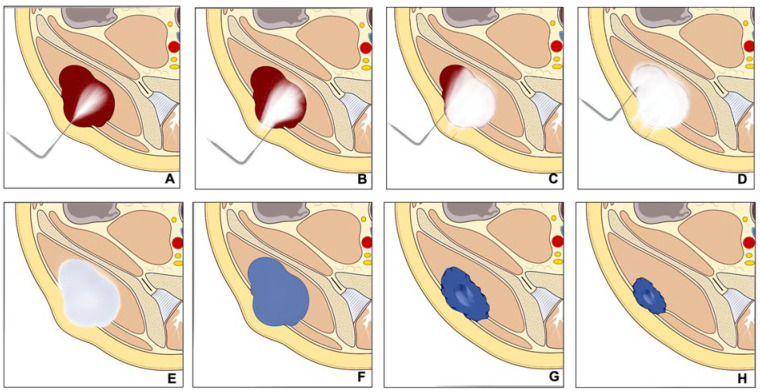
Sequence of events showing the cryoablation technique. The cryoprobes are inserted inside the lesion (**A**–**D**) until it is all covered by ice (**E**). The cooling damages cells causing ischemia and devascularization, which result in lesion destruction and volume reduction (**F**–**H**).

Cryoablation is a minimally invasive procedure that uses one or more probes filled with compressed gas (usually argon) to cool tumor tissue to very low temperatures [5]. This cooling is achieved by exploiting the Joule–Thompson effect, where rapid decompression of the gas surrounding the probe tip leads to temperatures below −20 °C [6,7]. The cooling damages the cells by forming intracellular ice crystals that destroy them, as well as impairing the blood supply by damaging the endothelial cells, which results in local ischemia and devascularization (Figure 1). Its integration with CT (Computed Tomography) technology allows for more accurate targeting of tumor cells, reducing collateral damage to surrounding healthy tissues, and minimizing postoperative complications [3,8,9].

This article aims to report on the use of CT-guided cryoablation in treating bone and soft tissue tumors, including sarcoma, metastases, and benign primary tumors, and the potential benefits and challenges associated with this novel therapeutic approach, including recent technical developments in cryoablation techniques. We will explore current research and clinical studies, highlighting the impact of this technique on cancer treatment and patient quality of life, and its promising role in the future of musculoskeletal oncology.

## 2. Malignant Bone and Soft Tissue Tumors

Percutaneous cryoablation is becoming an increasingly accepted option within the multidisciplinary sarcoma board for the treatment of primary bone and soft tissue tumors, applicable for selected cases. Despite surgical intervention being the mainstay for treating primary, non-metastatic bone, and soft tissue tumors, the local control of recurring bone and soft tissue sarcoma (STS) continues to be a challenging task. It mainly hinges on the disease prognosis as per the guidelines of the European Society for Medical Oncology (ESMO) [10,11]. Surgical resection is the common protocol for localized conditions, while chemotherapy or radiation therapy may be employed for more extensive diseases or recurrences [10,11,12]. Lately, minimally invasive techniques such as radiofrequency ablation, microwave ablation, or cryoablation have been proposed as potential surgical alternatives for some selected recurrent bone and soft tissue tumors [13,14,15,16,17]. Studies evaluating the role of cryoablation in the management of malignant bone and soft tissue tumors are summarized in Table 1.

Some initial studies evaluated the therapeutic effect of Cryoablation for the treatment of a variety of primary bone and soft tissue malignancies with promising results; however, the scientific evidence is still limited. Moreover, there is a recognized need for the standardization of selection criteria for percutaneous cryoablation. Lippa et al. [12] aimed to identify these criteria, finding high agreement for all proposed criteria between two readers. Eligibility for cryoablation was significantly associated with tumors located deeply, with great axes ≤ 5 cm, high local tumor aggressiveness, and a diagnosis of differentiated myxoid liposarcoma or myxofibrosarcoma.

### 2.1. Recurrent Retroperitoneal Soft Tissue Tumors

Some retrospective studies have reported on the effectiveness and safety of percutaneous cryoablation in the treatment of recurring retroperitoneal soft tissue sarcomas (RPSs). RPSs, which make up approximately 0.15% of all cancers, originate in the retroperitoneum but not from its main organs. Their proximity to critical structures makes them challenging to manage. Surgery is the primary therapeutic approach for localized cases, leading to a survival rate of about 60% over five years [21,23]. Nonetheless, complications with the removal of RPSs can impact survival and lead to recurrence. For recurrent cases, re-operation is recommended, but it is more difficult, and the additional benefits of chemotherapy and radiotherapy are debatable. In some selected cases, cryoablation has proven safe and effective as a palliative treatment for RPSs and could be included in the armamentarium of the sarcoma board. Fan et al.’s retrospective analysis of data from 72 patients [21] primarily noted grade 1 and 2 adverse events, with fever being the most common, particularly in the group with larger tumors. The study found a median progression-free survival (PFS) and overall survival (OS) of 37.0 ± 7.7 months and 43.0 ± 5.9 months, respectively. A significant association was observed between tumor size and both PFS and OS, with smaller tumors linked to longer survival. No significant variance was seen in response rates between small and large tumor groups. Another study by Fan et al. found that percutaneous cryoablation significantly alleviated local pain in patients with recurring RPSs, with immediate relief more common in the group with smaller tumors [22].

### 2.2. Sacrococcygeal Tumors and Chordoma

**Figure 2:**
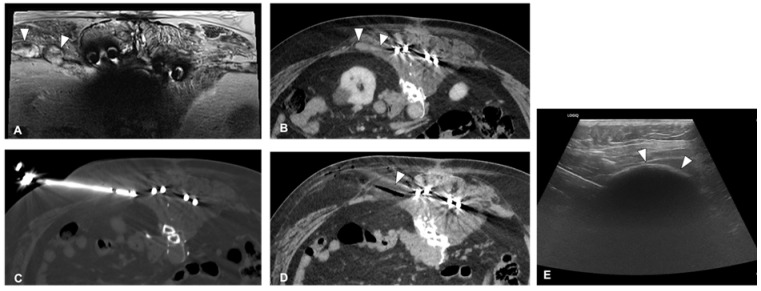
A 55-year-old man, cryoablation in a recurrent chordoma showed in an axial T2 MR image ((**A**)—arrowheads) and axial postcontrast CT image ((**B**)—arrowheads), for local tumor control. The cryoprobe was placed into the lesion under CT (**C**) and US guidance, with subcutaneous hydro dissection performed to protect the skin. The ice ball encompassed the entire lesion (**D**). Also, note the aspect of the ice ball on ultrasound ((**E**)—arrowheads).

Kurup et al. [20] documented the use of cryoablation in treating recurrent sacrococcygeal tumors, with the findings suggesting that this method was secure and relatively effective for local management or pain reduction (Figure 2). Similarly, Susa et al. [18] assessed the clinical outcomes of CT-guided cryoablation for recurring or metastatic malignant bone and soft tissue tumors, involving nine patients over a median observation period of 24.1 months. Although they reported promising outcomes, the study’s effectiveness was significantly limited by the small patient group. Additionally, Li et al. [19] recorded favorable results following the application of CT-guided argon–helium cryoablation in the treatment of sacral chordoma. All the patients achieved a complete response (CR), and there was a considerable improvement in chordoma-related symptoms post-treatment. The average score on the visual analog scale improved from 7.3 before treatment to 4.2 post-treatment, and the median progression-free survival was 36.8 months.

## 3. Bone Metastases

Bone frequently becomes a site of metastases, ranking as the third most common area for metastatic carcinoma after the lungs and liver [24]. Given the typically low survival rates of patients, therapeutic options are usually limited, and surgical resection is rare [24,25]. However, skeletal complications such as severe and chronic pain, spinal cord compression, and pathological fractures can greatly impair a patient’s quality of life [26,27,28,29,30,31,32]. The escalating interest in thermal ablation techniques for handling bone metastases has positioned cryoablation as a top-tier approach. This is due to its capacity to treat extensive and irregularly shaped pathological tissues in real time, while causing less pain compared to heat-based ablative methods like radiofrequency and microwaves [33,34,35]. The current body of literature mostly explores the palliative effect and local tumor control of cryoablation for bone metastases, which makes this technique a useful tool in the multidisciplinary management of cancer patients.

Studies assessing the role of cryoablation in managing bone metastasis are outlined in Table 2.

### 3.1. Pain Palliation and Disease Control

**Figure 3:**
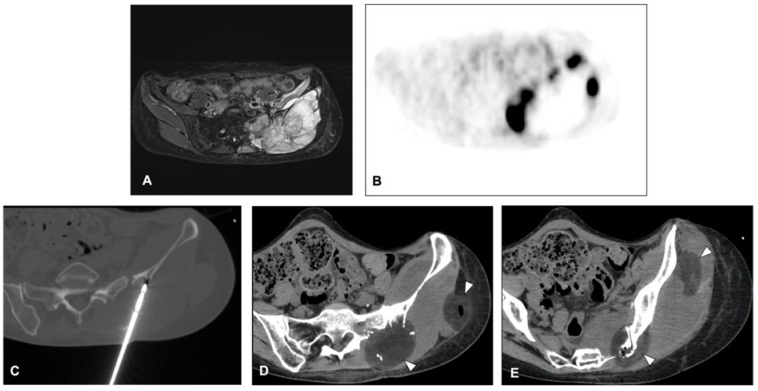
Axial T2 MR image of a 51-year-old woman with metastases from leiomyossarcoma (**A**) in the pelvis treated with cryoablation (**C**) for palliative intent. 18F-FDG PET/CT scan performed before the procedure demonstrates the pathologic radiotracer uptake in areas of viable tumor (**B**). The procedure was performed in an attempt to cover these areas and the post-procedure ice balls are visible as hypodense circles (arrowheads—(**D**,**E**)).

The clinical effect in pain reduction and safety of cryoablation for metastatic bone disease has been investigated by several studies in recent years [40,43,44,46,48] (Figure 3). A recent multicenter prospective study by Jennings et al. [38] assessed the clinical efficacy of cryoablation as a pain palliating method for patients with metastatic bone disease. The main goal was the pain score change from pre-treatment to the eighth-week follow-up, with participants monitored for 24 weeks post-treatment. A cohort of 66 participants (average age 60.8 years, 53% male) was recruited and underwent percutaneous cryoablation; 65 completed the follow-up. The average change in pain score from baseline to the eighth week decreased by 2.61 points. Average pain scores improved by 2 points at the first week and attained clinically significant levels (a decrease of more than 2 points) post the eighth week, with scores continuing to improve throughout the follow-up period. Quality of life was enhanced, opioid doses were steady, and functional status remained unchanged over six months. Severe adverse events were reported in three participants. Overall, the study suggested that cryoablation of metastatic bone tumors provided quick and long-lasting pain relief, enhanced quality of life, and presented an alternative to opioids for managing pain. The procedure was generally well received, and the severe adverse events were not directly linked to the procedure.

In a similar way, Arrigoni et al. [37] reviewed the results of 28 cryoablation procedures conducted over a span of seven years in their radiology department. These procedures included 17 palliative treatments and 11 curative treatments. A follow-up study after three months showed a substantial decrease in pain, as indicated by the average Visual Analog Scale (VAS) scores declining from 6.9 to 3.5. In patients treated for local tumor control, the study showed either stability or shrinkage of the lesion in 10 out of 11 patients, with no significant complications recorded.

In a prospective trial analysis, Callstrom et al. [52] evaluated the safety and effectiveness of cryoablation in pain reduction, enhancement of daily activities, and decreased usage of analgesics in patients with painful metastatic bone lesions. All eight patients who were on narcotic medication before the procedure reported a decrease in these medications after cryoablation, with no serious complications noted.

While useful for pain palliation and local tumor control, cryoablation of bone metastases could be used with curative intent in selected cases. Cazzato et al. [49] performed a retrospective review of patients who underwent cryoablation or radiofrequency ablation of bone metastases with curative intent. They observed noteworthy rates of local progression-free survival (LPFS), especially for bone metastases smaller than 2 cm. In addition, Autrusseau et al. [36] highlighted the long-lasting effect of cryoablation in treating extraspinal thyroid cancer bone metastases. The research was conducted on 16 patients with 18 bone metastases who underwent cryoablation from 2010 to 2020. Local tumor progression-free survival rates for the 1st, 2nd, 3rd, 4th, and 5th years were 93.3%, 84.6%, 76.9%, 75%, and 72.7%, respectively. Out of the 16 patients, 2 (12.5%) died during the follow-up period at 43 and 88 months. The significant adverse event rate was 5.5% (1 out of 18), with one instance of a post-ablative acromion fracture. The research concluded that cryoablation for extraspinal thyroid cancer bone metastases demonstrated high local tumor control rates and a generally safe profile in a long-term follow-up.

Moreover, De Marini et al. [50] conducted a comparison of the safety profiles of radiofrequency ablation (RFA) and cryoablation (CA) in the treatment of bone metastases. The study was conducted on 274 patients (average age 61.6 years) treated between January 2008 and April 2018. From these, 53 patients (involving 66 bone metastases) received RFA, and 221 patients (involving 301 bone metastases) underwent CA. Within the entire group, 2.5% of bone metastases resulted in major complications, with no significant discrepancy between RFA (1.5%) and CA (2.7%). However, RFA demonstrated a higher incidence of minor complications, predominantly post-procedure pain, at 33.3% compared to CA at 6.0%. The study concluded that RFA and CA have comparable low rates of major complications in treating bone metastases. Yet, RFA seems to lead to more post-procedure pain than CA, indicating the necessity for specialized pain management strategies for patients receiving RFA.

Finally, a recent systematic review [55] aimed to evaluate the safety and effectiveness of cryoablation in addressing painful bone metastases in cancer patients. The findings showed that cryoablation significantly diminished pain in patients with bone metastases, from one day to six months post-procedure. The largest average difference between pre- and post-procedure pain scores was 5.8 (on the VAS scale) at four weeks post-treatment. Moreover, cryoablation also enhanced the quality of life for these patients and decreased their reliance on painkillers. The spine was identified as the most commonly treated location. The procedure was generally safe with a combined minor and major complication rate of 12.74%.

### 3.2. Application to Spinal Metastases

The treatment of spinal metastases is notoriously difficult due to the sensitive nature of the spine, with both watchful waiting and active treatment carrying significant risks of local complications [56,57]. Cryoablation for spinal metastases is often conducted alone or frequently in combination with vertebral augmentation techniques such as cementoplasty [51]. In a study by Autrusseau et al. [51], 31 patients (including 36 spinal metastases in 32 sessions) received cryoablation for pain relief, and 10 patients (10 metastases in 10 sessions) for local control. The procedure successfully alleviated pain in 93.8% of palliative sessions, with the average pain scores notably decreasing at 24 h, 1 month, and at the final follow-up (approximately 16.5 months). For those patients needing local tumor control, primary clinical success was achieved in 60% of cases with about 25 months of median follow-up. The overall complication rate was 8%, with no reported secondary fractures or thermal nerve injuries. Tomasian et al. [41] previously reported similar results, reporting significant reductions in patient pain levels and requirements for pain medication at all evaluated follow-ups. Local control was achieved in 96.7% of tumors (30 out of 31) following an average follow-up period of 10 months. Only two patients reported transient post-procedural complications, specifically unilateral lower limb radiculopathy and weakness.

Similarly, Cazzato et al.’s 12-year retrospective study [45] from May 2008 to September 2020 involved 74 patients (35 women; median age 61) with 105 spinal metastases. Cryoablation was combined with cementoplasty in 72.4% of these cases. Out of 105 cases of spinal metastasis, 9 complications occurred (8.5%), 2 of which were major and 7 minor. Among the 86.5% of patients suffering from painful spinal metastases, the mean pain score dropped from 6.8 to 4.1 within 24 h, to 2.5 after one month, and to 2.4 at the last follow-up (approximately 14.7 months). For patients undergoing cryoablation with a curative aim, local tumor control was achieved in 82.1% of cases at an average follow-up of around 26 months. The study concludes that cryoablation, often used in conjunction with vertebral augmentation, is a safe and effective treatment for spinal metastases, offering quick and sustained pain relief and high rates of local tumor control at a 2-year follow-up. These studies suggest that cryoablation is not just safe, but also effective, yielding rapid and lasting pain alleviation and high rates of local tumor control.

### 3.3. Application to Sternal Metastases

Due to its high success rate and the safety provided by real-time visualization of the ice formation, cryoablation can be employed to treat highly sensitive body areas. A study by Hegg et al. [42] sought to evaluate the safety and efficacy of cryoablation for sternal metastases. The retrospective review included 12 patients with 12 sternal metastases. The results indicated that cryoablation provided pain relief, as shown by a drop in average pain score from 7.0 to 1.8. Local tumor control was achieved in 80% of the patients treated for this purpose. The study concluded that cryoablation is a safe and potentially effective treatment for painful sternal metastases.

When treating particularly sensitive areas like the sternum, certain thermal protective measures have been documented. Autrusseau et al. [58] assessed the use of retro-sternal space hydro dissection during sternal cryoablation as a method of protecting the pericardium. The findings demonstrated a technical success rate of 100%, indicating that the complete dual freeze protocol was executed without any contact between the ice formation and the pericardium in all instances. The hydro dissection procedure led to a significant increase in the minimum distance from the lesion to the pericardium, expanding from an average of 5.8 ± 3.8 mm prior to hydro dissection to 22.2 ± 5.8 mm post-procedure. The final ice formation was approximately 11.6 ± 8.7 mm away from the pericardium. An average volume of 198 ± 79.8 mL of iodinated contrast was injected for the hydro dissection. No immediate, short-term, or medium-term complications were reported. These results suggest that hydro dissection effectively moves the pericardium away from the ablation zone, offering thermal protection during sternal cryoablation.

### 3.4. Evaluation of Post-Ablation Area

Evaluating the treated area after cryoablation is crucial in determining the success of the procedure and detecting any local tumor recurrence. In this regard, Gravel et al. [53] conducted a study to assess the effectiveness of post-ablation MRI in identifying cases of incomplete treatment of spinal osseous metastases following cryoablation. The study involved 54 spinal bone metastases in 39 patients. The classification of MRI images into four categories resulted in a sensitivity of 77.3% and specificity of 85.9% in identifying residual tumors. The study concluded that post-cryoablation MRI is beneficial in assessing the effectiveness of the treatment, and proposed a classification system for post-ablation imaging.

### 3.5. Technical Consideration for Neuroprotection

Preserving neural structures is paramount when treating lesions close to the spine or major peripheral nerves. Kurup et al. [59] explored the use of motor evoked potential (MEP) monitoring during the cryoablation procedure of musculoskeletal tumors to reduce the likelihood of nerve damage. This study included 59 procedures on 64 tumors in 52 patients, with tumors located in various sites such as the spine, sacrum, retroperitoneum, pelvis, and extremities. During these procedures, MEP monitoring identified significant decreases in MEPs in 32% of the cases, with transient decreases in 25% and persistent decreases in 7%. Out of the four patients with persistent decreases in MEPs, two experienced motor deficits post-ablation. Conversely, no motor deficits were noted in patients with transient MEP decreases or no MEP changes. The study concluded that persistent reductions in MEPs are linked with post-procedure motor deficits, indicating that MEP monitoring during the procedure could help forecast neural damage, thereby increasing patient safety during the cryoablation of musculoskeletal tumors.

### 3.6. Technical Consideration for Bone Reinforcement

When conducting ablation on large bone sections or bones that bear weight [47], bone reinforcement might be required to avoid post-procedural pathologic fractures. Combining cryoablation with cement stabilization has been reported as highly effective by several studies. Masala et al. [60] studied the efficacy of combining cryoablation and vertebroplasty (CVT) vs. vertebroplasty alone in 46 patients with a single vertebral metastasis. They used the Visual Analog Scale (VAS) and the Oswestry Disability Index (ODI) to measure pain levels and quality of life. Although both treatment groups showed a significant reduction in VAS and ODI scores, more notable improvements were observed in the CVT group at various follow-up stages, suggesting CVT as a safe, effective option for pain relief and disability improvement. Kurup et al. [61] examined the efficacy of balloon-assisted osteoplasty following percutaneous cryoablation in seven patients with unilateral periacetabular tumors. Their findings showed a technically successful procedure in all cases, minor cement leakage in two patients, a non-displaced fracture in one patient, and local tumor progression in one of five patients with imaging follow-up. This study concluded that this treatment approach appears feasible, safe, and effective for preventing fractures. In another study, Kurup et al. [62] investigated the outcomes of 37 patients with acetabular metastases treated with combined cryoablation and cementoplasty. The results indicated pain reduction in 85% of patients and revealed no significant difference in pain reduction between complete vs. incomplete ablations. However, patients with previous acetabular radiation therapy or surgery experienced increased fractures post-treatment. Similarly, Coupal et al. [39] evaluated the effectiveness and safety of image-guided percutaneous cryoablation and cementoplasty for palliating large pelvic bone metastases, finding a 100% technical success rate, no immediate complications, and a significant decrease in pain scores post-intervention.

Finally, another study assessed the combination of aledronic acid and cryoablation treatment for managing bone metastatic pain [54]. Eighty-four subjects were divided into three groups and underwent different treatment combinations. The results showed cryoablation led to a significant drop in pain scores at week 2, while zoledronic acid showed a delayed response at week 4. However, the combination of cryoablation and zoledronic acid showed superior efficacy in terms of rapid response and sustained pain control, with no serious adverse effects or complications reported.

### 3.7. Combination Treatment

Sundararajan et al. [63] proposed a sequential interventional therapy involving embolization, cryoablation, and osteoplasty for patients with osseous neoplasms, who were unresponsive to conventional treatments such as oral analgesia and radiotherapy. The results suggested a significant reduction in mean pain scores post-treatment and a decrease in oral analgesic requirement, implying the effectiveness of this combination therapy in palliation.

### 3.8. Complications

Despite cryoablation being a minimally invasive procedure guided by CT, it carries a small risk of complications. Auloge et al. [64] evaluated the complications and related risk factors in bone tumor cryoablation. The study involved 239 patients who underwent cryoablation for 320 primary or metastatic bone tumors from 2008 to 2017. The overall complication rate was 9.1%, with serious complications making up 2.5% of this total. The most common major complication was secondary fractures, which represented 1.2% of the cases. Additional complications ranged from infections, tumor seeding, and bleeding, to severe low blood pressure, postprocedural pain, peripheral nerve damage, and temporary abnormal skin sensations. Risk factors for complications were found to be an Eastern Cooperative Oncology Group performance status (ECOG-PS) over two, cryoablation of long bones, and the use of more than three cryoprobes. For severe complications specifically, the risk factors included being over 70 years of age and the use of more than three cryoprobes.

## 4. Benign Bone Tumors

### 4.1. Osteoid Osteoma

Osteoid osteoma (OO) is a small, benign tumor primarily found in the bones of young people and children. Even though it only accounts for approximately 10% to 12% of all benign bone tumors, it can significantly affect the quality of life, causing pain and bone deformity, especially in children [65,66,67]. The treatment for OO has seen a considerable evolution over the years. Traditional surgical removal was once the main treatment approach, but technological advancements have facilitated a shift toward less invasive methods like radiofrequency ablation (RFA) [68,69,70,71].

Meng et al. [72] conducted a study comparing the safety and effectiveness of percutaneous CT-guided cryoablation of OO to surgical curettage. Both treatment approaches reported a 100% technical success rate. However, patients treated with cryoablation spent significantly less time in the hospital than those undergoing surgery, and both groups showed notable improvement in postoperative Visual Analog Scale (VAS) pain scores.

**Figure 4:**
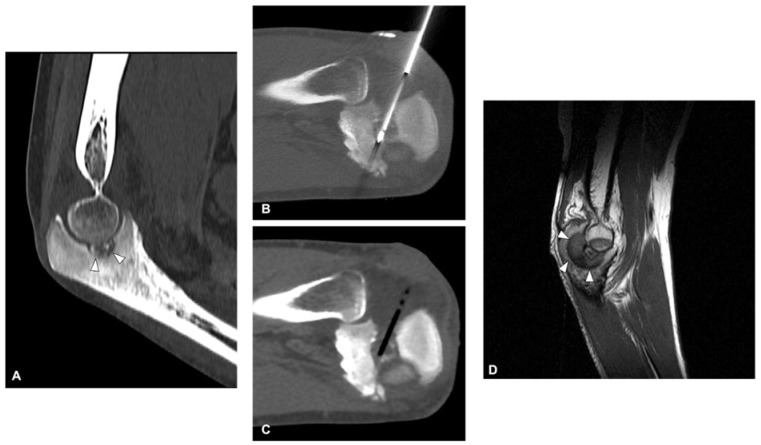
Cryoablation of osteoid osteoma of the elbow in a 28-year-old man: Coronal CT image shows the nidus (arrowheads) with surrounding sclerosis (**A**). Axial CT images acquired during the procedure show the cryoprobe placed inside the lesion (**B**) and the low-attenuation ice ball encompassing the lesion (**C**). Sagittal T1 MRI at 1-month follow-up shows signal change corresponding to the ablation area (arrowheads—(**D**)). Patient reported considerable improvement in pain.

Percutaneous cryoablation for OO treatment is increasingly being acknowledged as a safe and effective technique (Figure 4). Research assessing the role of cryoablation in managing osteoid osteoma is summarized in Table 3.

Two studies, one by Coupal et al. [73] and the other by Santiago et al. [74], were among the first to report the use of CT-guided percutaneous cryoablation in treating OO in adults. Coupal et al. reported a 100% clinical and technical success rate, with the average numeric pain scores decreasing significantly from 7.4 before the procedure to 0.3 at the follow-up. Similarly, Santiago et al. reported a substantial reduction in median VAS scores from 8 pre-procedure to 0 at the primary and secondary follow-ups. Both studies reported minor complications only, emphasizing the safety of the procedure.

The safety and efficacy of this procedure also extend to the pediatric and adolescent population, as shown in the study by Whitmore et al. [75]. It was found that immediate, short-term, and long-term clinical success was achieved in 96.4%, 96%, and 90.5% of patients, respectively. The median pain score dropped from 10 to 0 after the procedure. This study again highlighted that the procedure was technically feasible and had no major complications.

Miyazaki et al. [76] took a step further by conducting a prospective trial to assess the safety of percutaneous cryoablation for OO. They found no major complications associated with the procedure, while minor adverse events were observed in 22–67% of patients. The average Numeric Rating Scale (NRS) score significantly reduced from 7 before the procedure to 0 a year after treatment. These results suggest that cryoablation can be a safe and effective treatment for OO.

Percutaneous cryoablation has some key advantages over traditional OO ablation techniques like radiofrequency ablation. For instance, it allows the lesion to be treated by positioning the cryoprobe next to the bone cortex instead of penetrating the nidus. Additionally, cryoablation for OO can often be performed under local anesthesia and sedation because it tends to be less painful and more tolerable than radiofrequency or microwave ablation. These advantages were highlighted in a study by Le Coroller et al. [77] where cryoablation was carried out under local anesthesia and conscious sedation for 60% of the patients (30 out of 50). The cryoprobe was positioned outside the bone to cover the lesion, thus avoiding direct penetration of the nidus. The overall clinical success rate was 96% (48 out of 50 patients). Of the two patients who did not achieve clinical success, one had incomplete pain relief, and the other experienced a recurrence of the osteoid osteoma at 11 months. The latter was successfully treated with a second cryoablation procedure.

**Table 3:** Studies evaluating the role of cryoablation in the management of osteoid osteoma.

Author, Year	Reference	Number of Patients	Mean Age	Nidus Size (Mean)	NS/S	Success (%)	Follow-Up in Month	Complications	Results
Wu, 2011	[67]	6	12.6	6.3	6/0	100	28.7	None	Mean VAS was 6.57 ± 0.55 prior to the procedure and 0.57 ± 0.10 after 1 month.
Coupal, 2014	[73]	10	27.9	NR	10/0	100	24	None	Average pain scores were 7.4 before the procedure, 1.5 after the procedure, 0.5 at the primary follow-up, and 0.3 at the secondary follow-up.
Santiago, 2018	[74]	21	29.9	7.5	16/5	95.2	21	3 minor—1 mild skin burn, 2 soft tissue swelling and mechanical pain	Prior to the procedure, the median VAS score was 8 (range, 5–10), and at the primary and secondary follow-up, it was 0 (range, 0–2; *p* < 0.0001) and 0 (range, 0–7; *p* < 0.0001).
Whitmore, 2016	[75]	29	11.3	6.7	28/1	90.5	18.3	3 mild dermal blistering, 2 cases of weakness and pain, and 1 of transient numbness	19 of 21 patients (90.5%) experienced long-term clinical success (cessation of pain and NSAID use for 4 to 12 months following the treatment).
Miyazaki, 2018	[76]	9	20	5.9	7/2	100	11.7	No major; 5 pain	Before treatment, the mean NRS score was 7, and it was 0.6, 0.1, and 0 after 4 weeks, 6 months, and 1 year, respectively, after treatment.
Meng, 2021	[72]	15	16.1	14.6	12/3	100	>12	1 mild numbness of the lower extremity, 2 mild postoperative pain	All post-procedure VAS scores improved compared to the pre-procedure scores.
Cazzato, 2019	[78]	10	21	16.5	5/5	100	12	1 major—permanent sensory deficit of the arm; 1 minor—transient right Horner syndrome	At 1 and 12 months of follow-up, primary clinical success was 100% and 78%, respectively, with 2 patients presenting recurrent pain.
Le Corroller, 2022	[77]	50	24	6.0	41/9	96%	18–90	3 minor (2 transient pain and soft-tissue swelling and 1 mild skin burn)	Before the procedure, the mean VAS was 8, and after the procedure, it was 0 at both primary (6 weeks) and secondary (18–90 months) follow-up.

VAS: visual analogue scale; NRS: numerical rating scale.

### 4.2. Osteoblastoma

Cryoablation was also found to be a viable treatment for osteoblastoma in the study by Cazzato et al. [78] Technical success was achieved in all cases, and primary clinical success was 100% and 78% at 1 and 12 months of follow-up, respectively. Notably, this study emphasized the need for comprehensive protective measures due to the frequent close proximity of critical structures. In a similar vein, Serrano et al. conducted a retrospective review involving 11 pediatric and adolescent patients diagnosed with chondroblastoma and osteoblastoma. The study demonstrated both technical and clinical success in all patients, with no signs of recurrence observed on imaging follow-up. However, a single patient developed a transient radiculopathy as an immediate complication, underscoring the need for cautious application and vigilant follow-up.

### 4.3. Bone Cyst and Aneurysmal Bone Cyst

Bone cysts are fluid-filled holes that develop within bones. They are commonly found in children and adolescents, and most often occur in the long bones of the body such as the femur or the humerus. Most bone cysts do not cause symptoms and are often discovered incidentally during an X-ray performed for other reasons. However, in some cases, they can cause pain or lead to fractures [79].

Aneurysmal bone cysts (ABCs), on the other hand, are an uncommon type of bone cyst that is blood-filled rather than fluid-filled. They can occur at any age but are most commonly diagnosed in individuals under the age of 20. ABCs are expansile and can cause pain, swelling, and deformities in the affected bone. They can also lead to fractures due to the weakening of the bone structure. Although benign, ABCs are aggressive and can cause significant bone destruction if left untreated. For asymptomatic cysts, an initial wait and see is advised and intervention is recommended in case of progression or beginning of symptoms. They are typically treated through surgical intervention, but less invasive methods are increasingly being explored [80,81,82].

Alkuhaimi et al. [83] reported combined cryoablation and bone graft substitute treatment on six patients with symptomatic bone cysts. They reported a 100% technical success rate, with all patients showing bone cyst mineralization, reaching 80% at a median time of 6 months. Pain relief was achieved in all cases, with no major complications reported during the 31.5-month median follow-up period.

Arleo et al. [84] evaluated minimally invasive treatments, including cryoablation and doxycycline sclerotherapy, for aneurysmal bone cysts in 21 patients. Major complications occurred in 7.7% of procedures. On average, patients required fewer procedures with cryoablation (average 1.7) compared to doxycycline sclerotherapy (average 3). Follow-up imaging showed improvement in 85% of patients, and 93.8% reported reduced or absent pain.

## 5. Desmoid Tumors

**Figure 5:**
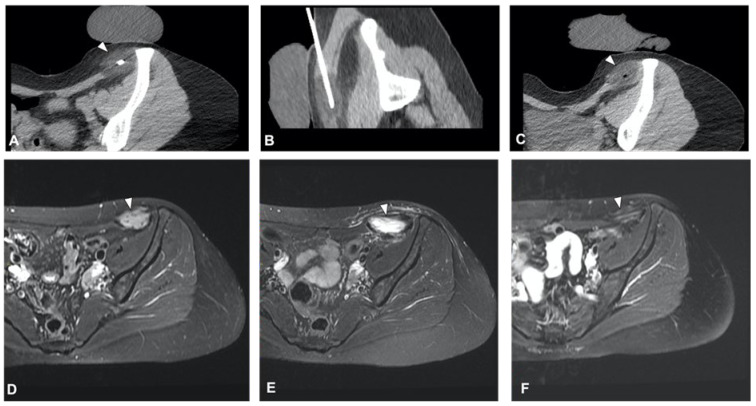
A 46-year-old patient with desmoid tumor of the abdominal wall showed in axial T2 fat sat MR image ((**D**)—arrowhead). Axial (**A**) and sagittal oblique (**B**) CT images showing the cryoprobe inside the lesion and the post-procedure ice ball containing the whole lesion (**C**). Axial T2 fat sat MR image 3 months after treatment shows an increase in T2 signal inside the lesion, suggesting necrosis (**E**), and the control with 6 months shows no residual tumor (**F**).

Desmoid tumors are rare benign tumors originating from musculoaponeurotic structures [85]. Despite their benign nature, they can display local aggressiveness, causing disability and sometimes pain. The ESMO advises initial observation and subsequent medical treatment for progressing tumors. Cryoablation, an interventional radiology technique, is recommended for desmoid tumor patients due to its ability to induce cell death through multiple cycles of freezing [86,87,88] (Figure 5).

Given the high recurrence rate, surgery’s role in treating desmoid tumors is restricted only to selected cases [89]. However, percutaneous cryoablation has recently come into the spotlight as a promising treatment option, demonstrating positive results in safety, effectiveness, and symptom alleviation [90,91,92]. A systematic review and meta-analysis conducted by Vora et al. [93] scrutinized the safety and effectiveness of cryotherapy in treating extra-abdominal desmoid tumors. The findings suggested that the combined estimated percentage of major and minor complications was 4.2% and 10.2%, respectively, signifying a relatively low risk. The rate of disease non-progression across all the studies was significantly high at 85.8%. The one-year and three-year progression-free survival rates stood at 84.5% and 78.0%, respectively. Regarding pain management, the study discovered that 87.5% of patients who initially had a Visual Analog Scale (VAS) score of three or more experienced a reduction in their VAS score by at least three points. Furthermore, between 37.5% and 96.9% of patients reported experiencing partial or complete relief of symptoms. The study definitively concluded that cryotherapy is a safe and effective treatment for extra-abdominal desmoid tumors, providing short to medium-term effectiveness, comparable to traditional treatments. Moreover, another systematic review by Cazzato et al. [5] emphasized the benefits of considering cryoablation as a first-line treatment, suggesting it could potentially enhance clinical outcomes. A summary of studies exploring the role of cryoablation in managing desmoid tumors is provided in Table 4.

### 5.1. Cryoablation for Disease Control

Mandel et al. [98] conducted a study aimed at comparing the outcomes of patients with primary and recurrent extra-abdominal desmoid tumors undergoing percutaneous cryoablation with those undergoing surgical treatment. In this study, 22 patients treated with cryoablation were compared with 33 surgical patients. The median monitoring periods were 16.3 months for cryoablation and 14.9 months for surgery. Local recurrence-free survival (LRFS) rates for two years stood at 59% for cryoablation and 71% for surgery, although the median LRFS for surgery was not met. Two-year disease control reached 85% for all patients, with no significant difference between the cryoablation and surgical groups. Notably, the study revealed that no local recurrences happened after the first cryoablation in patients who had none or one of the following risk factors: tumor size larger than 5 cm, age less or equal to 25 years, or a history of locally recurrent disease. In contrast, all patients presenting with two or more of these risk factors experienced local recurrence after the first cryoablation. The study’s main conclusion is that percutaneous cryoablation of primary and locally recurrent extra-abdominal desmoid tumors provides a satisfactory progression-free survival and long-term disease control, comparable to that of surgical intervention.

Another study by Yan et al. [88] assessed the safety and effectiveness of cryoablation in treating desmoid tumors over a decade. The results demonstrated that cryoablation was successful in shrinking the volume of the viable tumor, with a median change of −43.7%. The majority of patients exhibited a partial response (61.5%) or stable disease (30.8%). Symptomatic relief was attained in 96.9% of cases, and there was a low occurrence of major complications (2.4%). The authors concluded that cryoablation seems to be a safe and effective treatment for local control of extra-abdominal desmoid tumors.

### 5.2. Ablation Margin

Although technically benign tumors, desmoid tumors have a high potential for local recurrence and local invasion. For this reason, they should be treated as malignant lesions, and to obtain a curative effect, all the lesions should be covered by the ice ball, leaving some margins if possible. In this regard, Schmitz et al. [95] shared their extensive 10-year experience using percutaneous cryoablation for treating desmoid tumors. They reported no serious complications and significant shrinkage of 95.7% of the tumors treated. However, in all instances of residual or advancing disease, recurrence happened at the edge of the previously treated tumor.

### 5.3. Pain Reduction

One of the main symptoms of a desmoid tumor is local pain and discomfort. Bouhamama et al. [99] focused on cryoablation’s analgesic efficacy. This study emphasizes the analgesic effect of cryoablation in the treatment of desmoid tumors, reporting a significant reduction in pain post-procedure. It also reports a disease-free survival rate of 42.2% at 3 years, providing a more complete picture of the long-term efficacy of cryoablation.

Moreover, Tremblay et al. [96] underscored the high degree of symptom improvement and local tumor control following cryoablation, while also highlighting the relatively low morbidity associated with the procedure. This study echoed the findings of Schmitz et al. [95], but with a more explicit focus on the symptom relief provided by cryoablation.

In terms of treating advanced and refractory desmoid tumors, the study by Auloge et al. [94] suggested that cryoablation could be a viable solution. The researchers reported a considerable objective response rate (80%) and pain reduction (96.7%). These findings are particularly significant considering the study population consisted of patients with symptomatic tumors that were unresponsive to previous treatments.

The prospective study by Kurtz et al. [86], CRYODESMO-01, further solidified the position of cryoablation as a feasible treatment option for progressive DTs. The study reported an impressive non-progression rate of 86% at 12 months post-treatment, along with substantial improvements in functional status and pain scores.

### 5.4. Patient Selection

In contrast, Testa et al. [97] highlighted the trend toward active monitoring as the first line of treatment for desmoid tumors (DTs), reserving systemic and local ablative therapies for cases where the disease is advancing or causing symptoms. This study suggested that determining which patients are better suited for a primary non-interventionist approach as opposed to a direct interventional treatment like cryoablation remains uncertain. Patients with less serious and mildly symptomatic desmoid tumors were advised to begin with a strategy of active surveillance, and it was observed that tyrosine kinase inhibitors, local ablation, and surgery provided similar outcomes in patients with more aggressive conditions.

## 6. Technical Consideration

### 6.1. Planning and Approach

Cryoablation planning requires pre-procedural imaging, ideally within a month prior to the procedure. Thin-section CT is recommended for bone lesions, while MRI with dynamic contrast enhancement and high spatial resolution techniques offer higher sensitivity and specificity for soft tissue lesions [100].

### 6.2. Needle Placement and Hydro Dissection

When the lesion is not deeply located and is outside the bone, as in soft tissue tumors, the ultrasound-guided placement of the needles may expedite the process and reduce patient exposure to ionizing radiation [100,101,102]. Hydrodissection is a valuable technique, used to create a safe margin between the tumor and adjacent critical structures. To enhance visibility during control CT scans, it is recommended to use iodinated water for hydro dissection. This allows the operator to clearly delineate the dissected area [58].

### 6.3. Ablation and Monitoring

During the ablation phase, real-time visualization of the ice ball using ultrasound can assist in limiting the number of CT scans. However, the complete ice ball cannot be fully visualized due to shadowing; therefore, visualization through CT is essential [103]. When performing cryoablation near neural structures, such as the spine or peripheral nerves, continuous monitoring of evoked potentials is necessary. If a decrease in potentials is observed, the procedure should be halted immediately, as the ice ball may continue to grow for several minutes after the cycle’s interruption. This strategy aids in preventing neural damage, a significant potential complication of cryoablation [59].

## 7. Discussion

The current body of literature extensively examines the application of percutaneous cryoablation for various types of malignant and benign bone and soft tissue tumors. The flexibility and precision of this treatment method, combined with its relative safety, make it a promising option for a variety of tumor types and sizes.

Regarding treatment effectiveness and safety, the visualization of the ice ball offers high precision. It permits the simultaneous use of many cryoprobes, allowing for the ablation of large lesions (>5 cm), the creation of ice balls with a diameter greater than 8 cm, and the shaping of the ablation zone to the shape of the target lesion through varied geometry of probe placement. In general, when multiple probes are used, they are placed approximately 2 cm apart within the tumor and 1 cm from the outer tumor margin. It is also less painful than RFA during ablation and after treatment, with a shorter hospital stay [104,105].

For primary bone and soft tissue tumors, the work by Lippa et al. [12] underscores the necessity of standardized selection criteria for eligible patients. Their study suggests that deep tumors with high local aggressiveness and a diameter ≤5 cm may be best suited for cryoablation, particularly in cases of differentiated myxoid liposarcoma or myxofibrosarcoma.

Cryoablation also shows considerable potential in managing recurrent retroperitoneal soft tissue sarcomas (RPSs). As observed by Fan et al. [21], adverse events associated with the procedure are predominantly minor, with fever being the most common. This safety profile positions cryoablation as a feasible alternative to surgery, which can often present more significant challenges and complications.

When it comes to the treatment of recurrent sacrococcygeal tumors and chordoma, research by Kurup et al. [20], Susa et al. [18], and Li et al. [19] points to the relative effectiveness and safety of cryoablation for local control or pain relief. However, the limited sample size in Susa et al.’s research [18] indicates a need for further study with more extensive participant groups.

Percutaneous cryoablation has also shown potential in the management of bone metastases. Even with typically low survival rates and complex treatment scenarios for these patients, cryoablation appears to be a beneficial approach for pain alleviation, local tumor control, and possibly even curative intent in certain situations [33,34,35,38,49,52,55]. However, Cazzato et al.’s [49] findings suggest that the best results might be achieved with bone metastases smaller than 2 cm.

Cryoablation also has a role in treating benign bone tumors. With respect to osteoid osteoma, studies by Meng et al. [72], Coupal et al. [73], and Santiago et al. [73] confirm the safety and efficiency of percutaneous cryoablation compared to traditional surgical procedures. Regarding osteoblastoma, the work by Cazzato et al. [78] underscores the importance of comprehensive safety precautions due to the common closeness of vital structures.

In treating symptomatic bone cysts and aneurysmal bone cysts, studies by Alkuhaimi et al. [83] and Arleo et al. [84] depict the technical success and beneficial outcomes of cryoablation. Still, Arleo et al.’s findings suggest fewer procedures might be required with cryoablation compared to other treatments, such as doxycycline sclerotherapy.

For desmoid tumors, which can display local aggressiveness, cryoablation has emerged as a promising technique [85,86,87]. Vora et al.’s [93] systematic review and meta-analysis support this, presenting cryotherapy as a safe and effective treatment with relatively low risk and high non-progressive disease rates. Mandel et al.’s [98] comparative study also suggests promising outcomes for patients who underwent cryoablation.

While these studies collectively establish a favorable safety profile and clinical efficacy for cryoablation in treating various tumors, it is essential to consider certain technical aspects for optimal results. The planning and execution of the procedure, including pre-procedural imaging, needle placement, and monitoring during ablation, are essential for a better outcome.

### Future Directions

One significant area for further investigation lies in the standardization of selection criteria for cryoablation candidates. Future studies should aim to define more precise eligibility factors, such as tumor location, size, local aggressiveness, and specific diagnoses. This would provide a framework to guide clinical decision making, ensuring the most suitable patients are selected for the procedure.

Future work should also explore the combination of cryoablation with other local or systemic treatments, especially for metastatic lesions. Given the promising results of cryoablation used in conjunction with cementoplasty, it would be interesting to research the use of cryoablation in combination with arterial embolization, radiotherapy, or immunotherapy.

Furthermore, more longitudinal studies and randomized controlled trials are needed to assess the long-term effectiveness and safety of percutaneous cryoablation. Despite the promising results, the majority of studies are retrospective and involve small patient cohorts, limiting the generalizability of findings.

## 8. Conclusions

Percutaneous cryoablation is a reliable and successful method for treating a range of bone and soft tissue tumors, being increasingly incorporated into the multidisciplinary decision-making process of tumor boards. The procedure’s benefits encompass prompt alleviation of pain, enhancement of life quality, and limited unwanted side effects. Precision in needle placement and ice ball visualization, facilitated by imaging guidance, further enhances the safety and accuracy of the procedure, making it a viable option even for lesions located near critical structures. Despite these benefits, the decision to perform cryoablation should consider tumor location, size, and aggressiveness.

Cryoablation has demonstrated substantial potential in handling a variety of musculoskeletal tumors, from retroperitoneal soft tissue sarcomas to sacral chordomas, bone metastases, and benign bone and soft tissue tumors, underlining its adaptability. However, more research is required to fully understand the extent of cryoablation’s capabilities. Future research directions should focus on establishing standardized patient selection criteria, examining the possibilities when cryoablation is paired with other localized or systemic treatments, and implementing more long-term studies and randomized controlled trials to evaluate its enduring effectiveness and safety.

## Figures and Tables

**Table 1:** Studies evaluating the role of cryoablation in the management of malignant bone and soft tissue tumors.

Author, Year	Reference	Tumor	Number of Patients	Tumor Size (Mean)	Mean Age	Follow-Up in Month	Complications	Results
Lippa et al., 2014	[12]	Local recurrence soft tissue sarcomas	13	4.8 cm	63.4	NR	2 (15.4%) minor, 4 mild (30.8%), 7 (53.8%) severe	Maximum diameter ≤ 10 cm, distance to skin > 5 mm, distance to neurovascular structures > 3 mm, absence of articular involvement, and planned cryoablation covering the entire lesion volume were used to determine eligibility for cryoablation. These criteria were assessed by two radiologists with perfect agreement (k coefficient 0.83 to 0.98).
Susa, 2016	[18]	Malignant bone and soft tissue tumors	9	NR	74.8	24.1	1 urinary retention, 1 transient nerve palsy, 1 minor wound complication	Median survival time was 35 months.
Li et al., 2020	[19]	Sacral chordoma	9	NR	53.6	33	NR	After treatment, the mean VAS score decreased from 7.3 to 4.2 (*p* = 0.001). Before treatment, the mean function score was 3.2; after treatment, it was 1.4 (*p* = 0.001). PFS was 36.8 months.
Kurup et al., 2012	[20]	Recurrent sacrococcygeal tumors	6	2 cm	58	15	1 minor—pain. No major complications	There was no sign of a recurrence in 4 tumors that had LTC treatment. For the two palliation patients, one had total pain relief (pain returned after 6 weeks), while the other had an instantaneous decrease in pain (from a score of 6 to a score of 2 on a scale of 10.
Lim et al., 2012	[16]	Bone tumors	32	NR	48	16.5	2 major—fractures	There were no recurrences in the primary bone tumor group. There were 3 cases of radiological relapses (*p* = 0.02) and 2 cases of clinical relapses in the metastases group. In the metastatic group, the median time for relapse-free survival was 17 months (*p* = 0.01).
Fan et al., 2016	[21]	Retroperitoneal soft tissue sarcomas	72	12.9 cm	49	NR	19 fever, 11 local pain, 10 emesis, 6 frostbite, 1 nerve injury	Mean PFS and OS were 37.0 ± 7.7 months and 43.0 ± 5.9 months. The difference in PFS and OS between the small tumor group and the large tumor group was statistically significant (*p* = 0.011 and *p* = 0.015, respectively), while the response rate (82.7% vs. 72.8%, *p* = 0.240) was not different.
Fan et al., 2016	[22]	Retroperitoneal soft tissue sarcomas	49	12.9 cm	50.3	18.5	fever (17), emesis (7), frostbite (5), local pain (4)	The median PFS was 13.4 months. At the end of follow-up, 13 patients had died and 26 were living. Significant differences between mean severe local pain scores in pre-treatment vs. post-treatment days were reported.
Ahlmann, 2007	[17]	Soft tissue sarcomas	38	NR	59	37	superficial wound infections (3), seroma (8), peripheral nerve palsies (5)	At 2 and 5 years, the DFS for patients with more than 95% necrosis was 85%. Disease-free survival for patients with less than 95% necrosis dropped to 60% at 2 years and 50% at 5 years.

VAS: visual analogue scale; PFS: progression-free survival; OS: overall survival; DFS: disease free survival.

**Table 2:** Studies evaluating the role of cryoablation in the management of bone metastases.

Author, Year	Reference	Pa/LTC	Median Tumor Size	Mean Age	Primary Tumor	Technique	Treatment Number	Follow-Up	Complications	Results
Autrusseau, 2022	[36]	LTC	1.9 cm	61	Thyroid cancer	CA	18	68 months	1 delayed fracture	Local tumor PFS at 1, 2, 3, 4, and 5 years was, respectively, 93.3%, 84.6%, 76.9%, 75%, and 72.7%.
Arrigoni, 2021	[37]	Pa, LTC	3–4 cm	62	Various	CA	28	3 months (Pa) and 22.4 (LTC)	1 grade 3 (bleeding)	LTC was 91% (10/11); mean VAS decreased from 6.9 (SD: 1.3) to 3.5 (SD: 2.6); *p* = 0.0001.
Jennings, 2021	[38]	Pa	NR	60.8	Various	CA	66	6 months	3 major—abdominal pain, hematoma, and skin burn or frostbite	Mean pain score reached clinically significant levels after 8 weeks.
Gallusser, 2019	[28]	Pa, LTC	NR	62	Various	CA (+5 long bone fixation)	18	12 months	1 delayed fracture	LTC was 63% (10/16); NRS score dropped significantly from 3.3 to 1.2 (*p* = 0.0024).
Gardner, 2017	[29]	LTC	3.4 cm	62	Renal cell carcinoma	CA	50	21.4 months	3 grade 3, 1 grade 4, and 5 delayed fractures	LTC was 82% (41/50).
Coupal, 2017	[39]	Pa	>5 cm	77.5	Various	CA	48	2.25 months	none	After the intervention, the mean pain score dropped from 7.9 to 1.2 (*p* = 0.001).
McArthur, 2017	[30]	Pa, LTC	NR	52.3	Various	CA	16	3 months	1 grade 1	Mean pain score improved for all patients; LTC was 93.8%.
Susa, 2016	[18]	LTC	3.9 cm	74.8	Various	CA	11	36 months	1 grade 1, 2 grade 2	2 patients had local recurrence.
Wallace, 2016	[40]	Pa, LTC	13 cm	53.9	Various	CA (+cementoplasty in 28% of the cases)	92	6 months	1 grade 1, 1 grade 3 (transient foot drop), and 2 grade 4 (hemothorax)	For 1 day, 1 week, 1 month, and 3 months, there were decreased median pain scores. LTC at 3 months was 90% (37/41); at 6 months it was 86% (32/37); and at 12 months it was 79% (26/33).
Tomasian, 2015	[41]	Pa, LTC	NR	53	Various	CA (1 cementoplasty and 1 vertebroplasty)	31	10 months	2 grade 1 (transient postprocedural radiculopathy and weakness)	LTC was 96.7% (30/31); at one week, one month, and three months, the numerical rating scale statistically significantly decreased (*p* = 0.001 for all).
Hegg, 2014	[42]	Pa, LTC	3.8 cm	57	Various	CA	12	5.7 months (Pa), 8.4 (LTC)	1 grade 2 (infection)	LTC was 80%; mean pain scores dropped from 7.0 +/− 1.9 at baseline to 1.8 +/− 1.2 (*p* = 0.00049).
Callstrom, 2013	[2]	Pa	4.8 cm	61	Various	CA	69	44 months	1 grade 3 (infection)	The mean pain score dropped from 7.1/10 to 5.1/10, 4.0/10, 3.6/10, and 1.4/10, respectively, at 1, 4, 8, and 24 weeks (*p* = 0.0001 for all).
McMenomy, 2013	[43]	LTC	2 cm	64	Various	CA	52	21 months	2 grade 3 (avascular necrosis and collapse of the femoral head and ureteral stricture)	LTC was 87% (45/52); 47 months was the median overall survival time; 1 and 2-year DFS rates were 7% and 22%, respectively. The median DFS was 7 months.
Ma, 2018	[44]	LTC	NR	NR	Non-small Cell Lung Cancer	CA	22	3, 6, and 12-month (LTC),1 month (Pa)	1 grade 3 and 1 grade 4 (pathologic fracture)	At 4 weeks of follow-up, there was a reduction in NRS pain scores from pre- to post-procedure (*p* < 0.01).
Cazzato, 2022	[45]	Pa, LTC	NR	61	Various		105	8 months	9 (2 major and 7 minor)	At the last follow-up, 34 patients (53.1%) reported being entirely pain-free. LTC was 82.1%.
Coupal, 2017	[39]	Pa	NR	77.5	Various	CA + cementoplasty	48	4.1 weeks (mean)	None	Following the intervention, there was a significant reduction in pain levels. Post-intervention pain ratings were stable for 1 to 9 weeks (mean: 4.1 weeks).
Yang, 2020	[46]	Pa	4.3 cm	67	Various	CA	36	20 months	3 complications (skin frostbite, nerve injury, pathologic fracture)	At 1 day, 1 month, 3 months, and 6 months following cryoablation, the response rates were 91.7%, 94.4%, 91.7%, and 94.4%, with CR occurring in 22.2%, 41.7%, 36.1%, and 22.2% of cases, respectively.
Susa, 2016	[18]	Pa	NR	78.7	Various	CA	11	24.1 months	1 case of urinary retention in a patient with sacral chordoma, 1 transient femoral nerve palsy	At the final follow-up, 4 patients had no signs of the disease, 2 were still living with the disease, and 3 died of the disease.
Prologo, 2014	[47]	Pa	1.7–12 cm	NR	Various	CA (+cementoplasty in 18 patients)	54	3 months	1 minor wound complication, 2 grade 1, 1 grade 3, and 3 grade 4	At 24 h and 3 months of follow-up, there was a significantly decreased median VAS and narcotic usage. (*p* < 0.000).
Prologo, 2014	[48]	Pa	NR	60.7	Various	CA	61	NR	2 minor complications and 4 major complications	* They only described cases with painful osseous metastatic/disease with adverse outcomes.
Cazzato, 2018	[49]	LTC	27.7 * RF and CA together	59	Various	CA	37	34.1	pain, partial anesthesia of buttock and difficulty urinating, fracture scapula, and seeding	28.5% of cases had local progression at the treated site; the 1- and 2-year LPFS were 76.8% and 71.7%, respectively. Local tumor growth was predicted by BM size (>2 cm) (*p* = 002). DFS at the same time interval was 86.3% and 61.5%.
De Marini, 2020	[50]	Pa, LTC	4.5 cm	61.5	Various	CA	301	19.5	18 minor and 8 major (4 fractures, 1 tumor seeding, 1 infection, 1 arterial bleeding, and 1 hypotension)	With RFA and CA of BM, similar low rates of major complications are expected. RFA seems to be more painful after procedure than CA.
Autrusseau, 2021	[51]	Pa, LTC	3.5 cm	59.7	Various	CA	41	16.5 (Pa) and 25 (LTC)	1 major—intraoperative cardiac arrhythmia 3 minor—pain, brachial plexus injury with spontaneous resolution, distended bladder	Clinical success was reached in 93.8% for palliation. At a median follow-up of 25 months, primary clinical success for LTC was achieved in 60% of spinal metastases.
Callstrom, 2006	[52]	Pa	1–11 cm	54	Various	CA	14	18	No major complications	The mean rating for the worst 24 h period of pain prior to cryoablation was 6.7; four weeks later, it was 3.8 (*p* = 0.003). Before therapy, the average level of pain interference with daily activities was 5.5 of 10, and it was 3.2 (*p* = 0.004) 4 weeks later.
Gravel et al., 2019	[53]	Pa	1.8 cm	54	Various	CA	54	12	2 grade 3 (1 persistent paraparesis and 1 Takotsubo cardiomyopathy) and 2 grade 2 (transitory radiculopathy with persistent dysesthesia in 1)	All 54 metastases had a 1-year complete treatment rate of 59.3%. The 1-year complete treatment rate per metastasis was 23 of 24 (95.8%) for lesions measuring less than 25 mm and farther than 2 mm from the spinal canal.
Staso, 2015	[31]	Pa	4–5 cm	69	Various	RT vs. CA RT vs. CA	25	3 months	1 humerus fracture, 7 injury to encased sacral plexus, 2 transient injury to adjacent peripheral nerve	In comparison to patients treated by radiotherapy alone (11.2%), a greater percentage of subjects treated with cryoablation (32%) or cryoablation followed by RT (72%;) experienced a complete response.
Mc Arthur et al., 2017	[30]	Pa	NR	52.3	Various	CA	16	436 days	short-term neuropraxia, which resolved within 48 h no major complications	After one week following the procedure and a 3-month clinical follow-up, all 16 patients reported improvement in pain. On follow-up CT scans, there was a total of 6.2% tumor growth and 93.8% tumor arrest or shrinkage, while all study patients progressed with non-cryoablated metastases at other sites in spite of systemic therapy.
Thacker et al., 2011	[32]	Pa	4.4 cm	60	Various	CA	36	24 h	1 temporary S1 vertebra dysesthesia 1 thermal injury over the ablation site	On a scale of 0 to 10, the pre-treatment pain scores for the two groups—6.5 for cryoablation and 6.0 for RFA—were not substantially different (*p* = 0.78). Following cryoablation, analgesic use in the first 24 h significantly decreased whereas following RFA, it increased (*p* = 0.03). Individuals who underwent cryoablation typically spent 2.5 fewer days in the hospital overall than individuals who received RFA (*p* = 0.003).
Li, 2014	[54]	Pa	5.8 (CA + ZA) 6.0 (CA)	51.8 (CA + ZA) 54.8 (CA)	Various	CA + zoledronic acid vs. CA vs. zoledronic acid	84	6 months	no major complications 1 frostbite, 8 pathological fractures	In the group receiving cryoablation treatment, the mean response of the worst and average pain considerably decreased at week 2 (all *p* = 0.05), whereas it did so at week 4 (all *p* = 0.05) in the group receiving zoledronic acid treatment. When compared to cryoablation, zoledronic acid treatments exhibited a more durable response to the worst and average pain between weeks 16 and 24 (all *p* = 0.05). In comparison to zoledronic acid alone, the cryoablation + zoledronic acid regimen significantly reduced the worst and average pain between weeks 1 and 4 (all *p* = 0.05) and had a longer-lasting effect on bone metastatic pain between weeks 12 and 24 (all *p* < 0.05) compared to cryoablation alone.

* PFS: progression free survival; VAS: visual analogue scale; NRS: numerical rating scale; LTC: local tumor control; DFS: disease-free survival; OS: overall survival; RFA: radiofrequency ablation; CA: cryoablation; BM: bone metastases; LPFS: local progression free-survival; RT: radiotherapy; CT: computed tomography.

**Table 4:** Studies evaluating the role of cryoablation in the management of desmoid tumors.

Author, Year	Reference	Number of Patients	Tumor Size	Mean Age	Follow-Up in Month	Complications	Results
Auloge et al., 2021	[94]	30	8.7 cm	39	18.5	4 major: 2 skin necrosis, 1 infection, and 1 brachial plexopathy; 7 minor: edema and temporary increase in pain	The PFS was 85.1% and 77.3% at 1 year and 3 years, respectively; 43% patients obtained a complete response, and 96.7% obtained a reduction in pain.
Kurtz et al., 2021	[86]	50	10 cm	41	12	31 grade 1; 29 grade 2; 15 grade 3, and 11 grade 4	At +12 months, the rate of non-progressing disease was 86%. Functional status and pain scores were significantly improved by cryoablation.
Yan et al., 2021	[95]	25	236.6 cm^3^	32	12	1 major: nerve palsy; 3 minor: pain, swelling	96.9% of patients achieved symptomatic relief. Symptomatic recurrence was 21.2% in the cohort (median time: 8 months). The median time for symptom improvement was 2.5 months.
Tremblay et al., 2019	[96]	23	6.9 cm	40.5	15.4	2 major: nerve injuries; 4 minor: hematoma, skin injuries	Symptomatic improvement was demonstrated in 89% of patients. At 12 months, the mRECIST response rate was CR 36%, PR was 36%, and SD was 28%. After cryoablation, no patients experienced rapid development of the residual disease.
Schmitz et al., 2016	[95]	18	6.4 cm	39.9	16.2	major: none; 3 minor: pain, emesis	In 9 of 23 tumors (39.1%), no residual viable EAD tumor was found; 22 of 23 tumors (95.7%) showed evidence of volume reduction; 1 of 23 tumors (4.3%) had progressive disease.
Efrima, 2021	[88]	11	258.6 cm^3^	35.3	48	minor: mild frostbite, limitation in range of motion, swelling	9/11 patients (82%) had a reduction in tumor volume and improved symptomatology. Tumor volume and viable segment reductions were, respectively, 36.7% (*p* = 0.0397) and 63.3% (*p* = 0.0477).
Havez, 2014	[91]	13	5.3 cm	39.5	11.3	1 major—transient peroneal nerve injury	DFS rate was stable at 6, 12, and 24 months at 82.3%. At 6, 12, and 24 months, the local tumor progression rate was 0%. Local recurrence was detected in 2 cases (12%).
Testa, 2022	[97]	20	NR	36.5	18	2 minor—pain at the ablation site	Active surveillance had a shorter 5-year PFS than cryoablation (*p* = 0.008). Patients who had cryoablation as their initial course of treatment had comparable 5-year PFS to patients who underwent surgery or surgery plus systemic therapy.
Saltiel, 2020	[92]	10	63.6 cm^3^	33	53.7	2 major—1 grade 3 (colo-cutaneous fistula) and 1 grade 4 (peroneal nerve palsy)	At 3, 6, and 12 months for patients who received curative treatment, the mean ET-V change was −97 ± 7%, −44 ± 143%, and +103 ± 312%, respectively. At 3, 6, and 12 months, the mean ET-V change for debulking patients was −98 ± 4%, +149 ± 364%, and +192 ± 353%, respectively.
Mandel, 2022	[98]	22	NR	NR	16.3	1 major—pneumothorax; 22 minor—pain	After cryoablation, the two-year local recurrence-free survival rate was 59%, and the median LRFS was 26.6 months.
Bouhamama, 2019	[99]	34	5.8 cm	38	6	2 hematoma grade 2; 2 grade 4 complications involving palsy of the common fibular nerve	At 3 years, DFS was 42.2%. The mean VAS was 5.7 and 2.4 at pre-treatment and 6 months, respectively. All measured tumor dimensions were lower than pre-treatment at 6 months.

PFS: progression-free survival; DFS: disease-free survival; ET-V: enhanced tumor volume; VAS: visual analogue scale.
